# Blood DNA methylation score predicts breast cancer risk: applying OPERA in molecular, environmental, genetic and analytic epidemiology

**DOI:** 10.1002/1878-0261.13117

**Published:** 2021-11-03

**Authors:** John L. Hopper, Tuong L. Nguyen, Shuai Li

**Affiliations:** ^1^ Centre for Epidemiology and Biostatistics University of Melbourne Melbourne Vic Australia

**Keywords:** breast cancer, DNA methylation, MEGA epidemiology, OPERA, polygenic risk score, risk prediction

## Abstract

In this issue, Kresovich and colleagues have published a hallmark paper in Molecular, Environmental, Genetic and Analytic Epidemiology. By applying artificial intelligence to the Sister Study they created a new methylation‐based breast cancer risk score (mBCRS) based on blood DNA methylation. Using a prospective design and after accounting for age and questionnaire‐based breast cancer risk factors, the Odds PER Adjusted standard deviation (OPERA) for mBCRS and polygenic risk score (PRS) was 1.58 (95% CI: 1.38, 1.81) and 1.58 (95% CI: 1.36, 1.83), respectively, and the corresponding area under the receiver operating curve was 0.63 for both. Therefore, mBCRS could be as powerful as the current best PRS in differentiating women of the same age in terms of their breast cancer risk. These risk scores are among the strongest known breast cancer risk‐stratifiers, shaded only by new mammogram risk scores based on measures other than conventional mammographic density, such as Cirrocumulus and Cirrus, which when combined have an OPERA as high as 2.3. The combination of PRS and mBCRS with the other measured risk factors gave an OPERA of 2.2. OPERA has many advantages over changes in areas under the receiver operator curve because the latter depend on the order in which risk factors are considered. Although more replication is needed using prospective data to protect against reverse causation, there are many novel molecular and analytic aspects to this paper which uncovers a potential mechanism for how genetic and environmental factors combine to cause breast cancer.

## Graphical abstract

This commentary highlights the introduction of a new DNA methylation‐based breast cancer risk score (mBCRS). Unlike genetic events, DNA methylation is an epigenetic process influenced by lifestyle factors, and changes the way the DNA code is read. The statistical approach called OPERA shows that mBCRS is as good as the current best genetic risk score in determining a woman’s breast cancer risk.

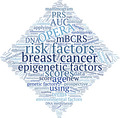

**Abbreviations**
AUC: area under the receiver operating characteristic curve
mBCRS: methylation‐based breast cancer risk score
MEGA: Molecular, Environmental, Genetic and Analytic
OPERA: odds ratio per adjusted standard deviation
PRS: polygenic risk score

In this issue, Kresovich *et al*. [1] have published a hallmark paper in Molecular, Environmental, Genetic and Analytic (MEGA) Epidemiology. The authors created a new methylation‐based breast cancer risk score (mBCRS) based on blood DNA methylation that could be as powerful as the current best polygenic risk score (PRS) in differentiating women of the same age in terms of their breast cancer risk.

The Sister Study had previously provided evidence that DNA methylation at individual sites is associated with breast cancer risk factors, but the evidence for a subsequent association with breast cancer risk *per se* has been inconsistent [2, 3]. DNA methylation changes with age, which has led to methylation‐based measures that predict age; the residual of these measures of so‐called biological age against actual age has been found to predict diseases, including breast cancer [4, 5]. Kresovich *et al*. have now made a further step, leveraging this information and resource and applying artificial intelligence to form mBCRS.

Using a prospective design and after accounting for age and questionnaire‐based breast cancer risk factors, the Odds PER Adjusted standard deviation (OPERA) [6] for mBCRS and PRS was 1.58 (95% CI: 1.38, 1.81) and 1.58 (95% CI: 1.36, 1.83), respectively, and the corresponding area under the receiver operating curve (AUC) was 0.63 for both. (Log(OPERA) is approximately linearly related to AUC‐0.5 in the range of AUC from 0.5 to 0.75.) This shows that the above epigenetic and genetic risk scores would be among the strongest known breast cancer risk‐stratifiers, shaded only by new mammogram risk scores based on measures other than conventional mammographic density, such as *Cirrocumulus* and *Cirrus*, which when combined have an OPERA as high as 2.3 (AUC = 0.72) [7]; see Table 1.

**Table 1:** Risk predictors for breast cancer classified by their odds ratio per adjusted standard deviation (OPERA) and equivalent interquartile risk ratio (IQRR).

Risk score	OPERA	IQRR
New mammogram risk scores combined	2.3	8
Multi‐generational family history scores	≤ 1.7	4
Methylation risk score (mBCRS)	1.6	3
Polygenic risk score (PRS)	1.6	3
Conventional mammographic density	1.4	2
High‐risk mutations (*BRCA1*, *BRCA2*)	1.2	1.5
First‐degree family history (yes/no)	1.2	1.5
Number of childbirths	1.1	1.3
Age at menarche	1.05	1.2
Age at first childbirth	1.03	1.1

Epigenetics is about how the action of genes depends on both the environment and the underlying genetic code. The DNA spells out the instructions, but epigenetic factors influence how these instructions play out in real life. This view of epigenetics challenges the concept of ‘genetic determinism’ and speaks to the way genetic and environmental factors combine, not compete, to determine disease risk.

The combination of PRS and mBCRS with the other measured risk factors gave an AUC of 0.71, which is equivalent to an OPERA of 2.2. If risk scores act independently, the squares of their log(OPERA)s should add. Therefore, combining the new mammogram risk scores with the epigenetic and genetic risk scores could give an OPERA as high as 3.1, or an AUC approaching the desirable threshold of 0.8 [8], a level of risk stratification, which would also be achieved if all the familial risk factors for breast cancer were known [9]. These OPERA and AUC are based on the assumption that the new mammogram risk scores act independently of both the PRS and mBCRS; our unpublished data find that the assumption for the PRS is not invalid, while the assumption for the mBCRS needs to be investigated.

The OPERA concept was devised to overcome a shortcoming of the AUC in making judgements about the risk predicting quality of a factor; the change in AUC depends on what is already in the model. OPERA uses the standard deviation of the risk score, which is the standard deviation of the risk measure after it has been adjusted for other factors involved in the design and analysis of a study. As Table 1 shows, the OPERA values of different factors can be compared, even across binary and continuous risk factors; this comparison could not be achieved using AUCs.

OPERA was conceived for case–control studies, and conceptually, its extension to cohort studies by using the hazard ratio seems simple. But prospective risk prediction typically involves age, given that a person’s disease risk typically changes with the advance of time. In terms of predicting events, knowing age is useful. In terms of deciding how good risk scores are in differentiating risk for persons of the same age, it is an impediment.

Also, publications on the performance of risk factors based on prospective data and using AUCs cannot be compared if they study different age distributions, or populations with different age‐specific incidences. OPERA overcomes this by requiring analysis of the risk factor distribution for the population for which inference is being made in terms of age. Kresovich *et al*. have come up with a clever way to address this issue using cohort data by applying sampling weights, and other strategies could also be used.

Application of OPERA requires an understanding of regression modelling beyond simply looking at estimates; finding optimal transformations for X and Y variables, being cognisant of the effects of outliers and influential points, calculating residuals from applying a formula developed using control or reference data to cases, etc. The PRS is a now familiar example of a normally distributed score across which risk increases multiplicatively. This might not be possible for all risk factors but OPERA‐ising a risk factor to have these nice properties, even approximately, is valuable for modelling, remembering that models are essentially untruths used in search of the truth. Supplemental fig. 8 of Kresovich *et al*. [1] suggests that there might be a subset of epigenetic probes that are even more strongly associated with risk, encouraging further research.

In terms of future directions, replication is essential and must be accomplished using prospective data to protect against reverse causation given that it is plausible that cancer, or more particularly its treatment, can cause changes in DNA methylation. Kresovich *et al*. have already replicated their findings using a case–control study nested in a cohort of Italian women. Further replication studies will be important to help create better risk scores and to address questions such as: does the strength of association differ by age, familial risk profile and other modifiers or risk? Is mBCRS familial and what are the causes for its familial aggregation [10]? Do nongenetic factors modify its risk association? What is its relationship to other breast cancer risk factors? And how does mBCRS combine with the PRS and the new mammogram risk scores?

We believe there are many novel molecular and analytic aspects to this paper, which uncovers a potential mechanism for how genetic and environmental factors combine to cause breast cancer.

## Conflict of interest

The authors declare no conflict of interest.

## Author contributions

JLH conceived the OPERA concept and TLN and SL were the first to apply it to mammogram‐based and methylation‐based risk factors. All authors contributed to the writing of this Commentary.
